# Optimization of the SiC Powder Source Material for Improved Process Conditions During PVT Growth of SiC Boules

**DOI:** 10.3390/ma12193272

**Published:** 2019-10-08

**Authors:** Oda Marie Ellefsen, Matthias Arzig, Johannes Steiner, Peter Wellmann, Pål Runde

**Affiliations:** 1 Fiven Norge AS—SIKA, Nordheim, 4792 Lillesand, Norway; paal.runde@fiven.com; 2 Crystal Growth Lab, Materials Department 6, Friedrich-Alexander Universität, 91058 Erlangen, Germany; matthias.arzig@fau.de (M.A.); johannes.steiner@fau.de (J.S.); peter.wellmann@fau.de (P.W.)

**Keywords:** SiC, source material, crystal growth, sublimation, in situ visualization

## Abstract

We have studied the influence of different SiC powder size distributions and the sublimation behavior during physical vapor transport growth of SiC in a 75 mm and 100 mm crystal processing configuration. The evolution of the source material as well as of the crystal growth interface was carried out using in situ 3D X-ray computed tomography (75 mm crystals) and in situ 2D X-ray visualization (100 mm crystals). Beside the SiC powder size distribution, the source materials differed in the maximum packaging density and thermal properties. In this latter case of the highest packaging density, the in situ X-ray studies revealed an improved growth interface stability that enabled a much longer crystal growth process. During process time, the sublimation-recrystallization behavior showed a much smoother morphology change and slower materials consumption, as well as a much more stable shape of the growth interface than in the cases of the less dense SiC source. By adapting the size distribution of the SiC source material we achieved to significantly enhance stable growth conditions.

## 1. Introduction

In recent years, SiC has become the most important semiconductor material for the fabrication of power electronic devices as they are mandatory in electromotive and energy-saving applications. Crystal growth of SiC is generally carried out using the physical vapor transport (PVT) method. Usually, SiC powder source material is sublimed at elevated temperatures above 2000 °C and crystallizes at a slightly colder seed. For a review on the SiC bulk growth process, refer to [1].

The proper choice of the SiC powder source during PVT growth is a prerequisite to achieve a high crystalline quality in the final SiC boule. In the literature, a number of factors that impact the growth process, like stoichiometry, purity, polytype, size distribution and related packaging density, have been discussed [2,3,4,5,6,7,8]. In principle, the ideal SiC source undergoes a minor morphological change during growth. The evolution should be smooth and the surface towards the crystal growth interface should be stable. The evolution of the source material and its impact on the crystal growth process have been investigated in a number of studies that make use of 2D and 3D X-ray-based in situ visualization [9,10,11,12].

SiC synthesis by the Acheson method has a long industrial history and has adapted to meet a changing market in applications. The versatility and energy efficiency compared with chemical routes make it an interesting alternative for SiC crystal growth by PVT. The growing demand for SiC power semiconductor devices will require high volumes of SiC source material that can still meet the strict technical requirements intrinsic to the applications. Acheson SiC could provide the market with quality SiC at high volumes and reduced cost.

The supply of large quantities of high quality SiC powder of high purity is eminent in order enable the large-scale application of SiC. The aim of this work is to study SiC source materials that have been synthesized by the high-volume Acheson process, subsequent to the powder synthesis, where purity and packing density have been optimized. The applicability of such a new SiC powder source has been investigated through 2D and 3D in situ X-ray visualization of PVT crystal growth experiments of SiC boules with a diameter of 75 mm and 100 mm.

## 2. Materials and Methods

### 2.1. Synthesis of the SiC Source Material

The SIKA High Purity Powder was synthesized using the Acheson process. In a customized furnace, high purity quartz sand and carbon black were reacted at high temperatures to form the crude. The material was then crushed and milled with specially developed equipment to minimize contamination.

The final size distribution was achieved by sieving with 106 µm and 450 µm sieves to remove the fine and coarse sides, respectively. Finally, the powder was treated chemically to remove impurities introduced during the processing steps.

### 2.2. Crystal Growth of SiC Boules

Crystal growth has been performed in two PVT growth reactors set up for SiC boules with a diameter of 75 mm and 100 mm, respectively. The furnaces exhibited a cylindrical double wall, and water-cooled side walls. The growth temperature was monitored at the top and at the bottom of the graphite growth cell by optical pyrometers. A numerical simulation using the software tool COMSOL Multiphysics (COMSOL Multiphysics GmbH, Göttingen, Germany) was carried out to determine the temperature distribution and gas composition inside the growth chamber.

### 2.3. In Situ Visulaization of the Growth Process

In situ visualization of the PVT growth process was performed using digital 2D and 3D X-ray-based imaging. Typically, up to ten images were acquired throughout one growth run that reveal the major sublimation and crystallization processes during one growth run. For details on the experimental X-ray imaging setup, refer to [9,12].

### 2.4. Characterization Methods

All powder characterization was done at the SIKA laboratory in Lillesand, Norway. The trace impurities were provided by the product datasheet for SIKA High Purity powder and measured by glow discharge mass spectrometry (GDMS) in an independent laboratory (Eurofins EAG, Toulouse, France). The particle size distribution was measured in the dry state by laser diffraction with a Malvern Scirocco 2000 (Malvern Panalytical, Malvern, UK). The total oxygen was measured with a LECO instrument (LECO Instruments, St. Joseph, MI, USA). Free carbon was determined according to ANSI B.74.15. Loose packed density (LDP) was measured according to FEPA standard 44. Micrographs of the powder were obtained using a Zeiss Evo MA10 (Zeiss, Oberkochen, Germany) scanning electron microscope (SEM) with secondary electron detection. The acceleration voltage was 15 kV and the working distance was 8.5 mm.

SiC wafer inspection was done at the FAU laboratory. Optical microscopy was performed using the devices Stemi 2000-C from ZEISS and Polyvar Met from Reichert-Jung, respectively. Birefringence measurements were taken in a homebuilt wafer mapping setup. Defect etching using KOH was carried out at ca. 510 °C in the setup described in [13,14]. Optical absorption in the visible and near infrared spectra was performed by a Perkin Elmer Lambda P950 UV/VIS spectrometer. Raman spectra were detected by Horiba Jobin Yvon LabRAM HR-800 spectrometer using a 633 nm excitation laser and a special resolution of ca. 1 µm. Photoluminescence was measured with 375 nm laser excitation using a Horiba Symphony IGA-512x1 detector.

## 3. Results and Discussion

### 3.1. Properties of the SiC Powder Sources

**Figure 1:**
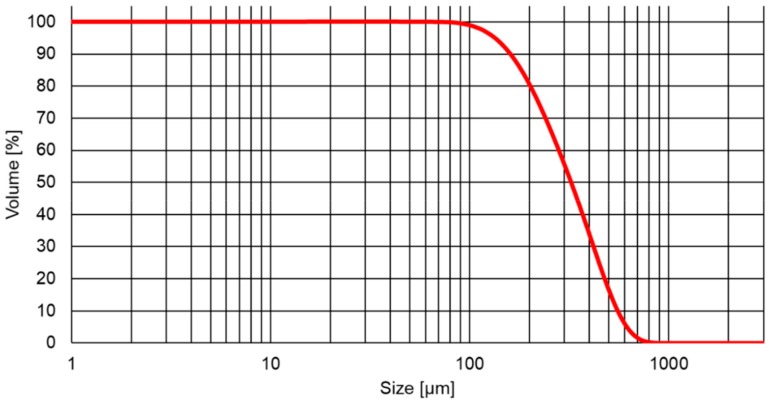
Particle size distribution of the SiC powder source SIKA High Purity SiC.

The size distribution and additional powder properties are presented in Figure 1 and Table 1. The particle size distribution of the SiC powder from SIKA shows a narrow size distribution, with a span of only 1.2. The mean diameter was 324 µm with a d90 and d10 of 160 µm and 555 µm, respectively. The LPD value was 1.6 g/cm^3^. The packing density of the powder along with high mean particle diameter are important factors to increasing efficiency and stability in the crystal growth process. 

The size distribution of the SIKA High Purity powder was developed to optimize the crystal growth process. The high packing density enables higher yield in PVT reactors. The large particle size and narrow distribution enables stable growth. Rounded grains would also improve stability during sublimation. The low free carbon and total oxygen contents are a result of the powder processing and are low compared with typical SiC products.

**Figure 2:**
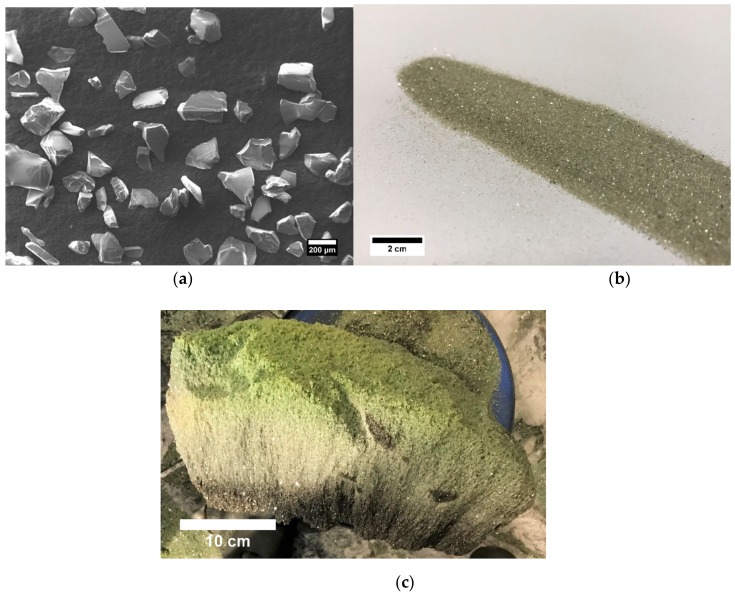
(**a**) SEM image of SiC powder. (**b**) Optical image of the SiC powder source. (**c**) Image of a piece of high purity SiC ingot before processing.

The SEM image of the powder in Figure 2a shows the grains were sharp, as is typical for SiC produced by the Acheson process. The optical image in Figure 2b shows the color of the powder is light green. Pure SiC is colorless, so the green tint is most likely due to nitrogen impurity. The nitrogen is introduced in the crystal during synthesis as the Acheson process is done in air. The image in Figure 2c depicts a piece of SiC ingot before processing, which clearly shows the range of colors that forms in the crude from white to dark green, corresponding to the diffusion of impurities through the furnace during synthesis. Sorting the material by color prior to processing helped increase the purity of the final product.

Table 2 presents a typical trace element analysis for the SIKA High Purity SiC powder. The total impurity is less than 17 ppm with metallic impurity below 14 ppm. The Acheson process and the raw materials used limit the purity that can realistically be achieved in a high capacity industrial process and therefore cannot compare to powders produced by CVD or other similar methods reaching 6N purity and higher.

### 3.2. Sublimation Behavior of the SiC Powder

**Figure 3:**
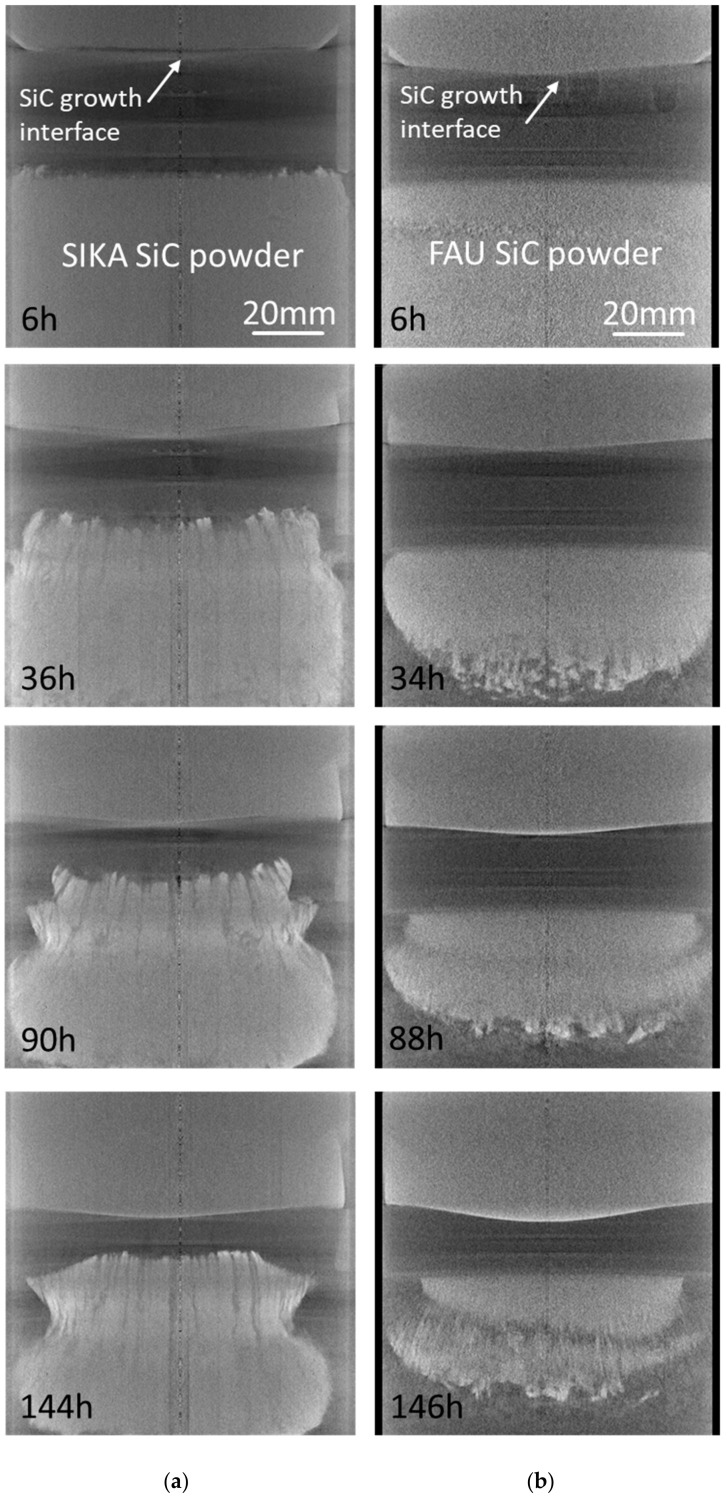
Series of 2D projections of the 3D in situ X-ray-based computed tomography visualization of the growth process of the 75 mm PVT growth process: (**a**) SiC powder from SIKA and (**b**) reference SiC powder from FAU.

Two SiC boules with a single crystalline diameter of 75 mm were grown using the SIKA and FAU SiC powder source for comparison. The average growth temperature and ambient inert gas pressure were set to 2050 °C and 20 mbar, respectively. Nitrogen gas was added in order to perform n-type doping. Simultaneously to the growth process, in situ 3D computed tomography X-ray visualization [12] was applied to study the sublimation behavior of the novel SIKA SiC powder source. Figure 3 illustrates, for both growth runs, the evolution of the SiC source material and SiC crystal growth process. In the case of the SIKA powder, in order to investigate the tendency of incorporation of carbon dust-like particles inside the growing SiC boule, no carbon dust shield was placed between source and seed. As a consequence, the source powder tends to rise up towards the crystal growth with progressing growth time. This trend is typical for all kinds of SiC source materials and not specific to the application of the SIKA powder. It can be suppressed in an optimized growth environment by marginal adaptions of the design of the hot zone. The formation of a needle-like structure on the top of the source material is observed, which is a typical behavior related to the concept of the applied growth setup (see, e.g., [9]). A significant difference between the SIKA and FAU powder is related to the difference in packaging density of 1.80 g/cm^3^ (SIKA) versus 1.15 g/cm^3^ (FAU). In the case of the much lower value for the FAU powder, a much faster shrinkage of the core of the SiC source material is observed, which causes a larger change of the growth conditions related to the temperature field. In the case of the SIKA source material, however, a more smooth and continuous consumption of the SiC powder is observed. Although the SIKA powder undergoes larger morphological changes in its top area because of the missing carbon dust shield, the grown crystal exhibits a much flatter growth interface which is beneficial to reduce thermo-elastic stress. The top area of the SIKA powder even tends to adapt with a slightly concave surface to the slightly convex crystal growth interface.

Optical analysis was carried out on a series of wafers cut from the SiC boule grown with the SIKA powder in order to investigate the occurrence of unwanted carbon dust particles from the SiC source. Within the resolution of the optical microscope of ca. 1 µm, no evidence for an incorporation of carbon particles from the SIKA powder into the grown SiC boule was found. In addition, KOH defect etching did not show an increase in the micropipe density that would be an indication of the presence of carbon particles even below the optical resolution. In both cases, the best areas of the SiC crystals grown by the FAU and SIKA powders exhibited a micropipe density below 5 cm^−3^. Areas with higher micropipe density were related to defective areas in the starting SiC seed wafer and are not related to the growth process itself.

**Figure 4:**
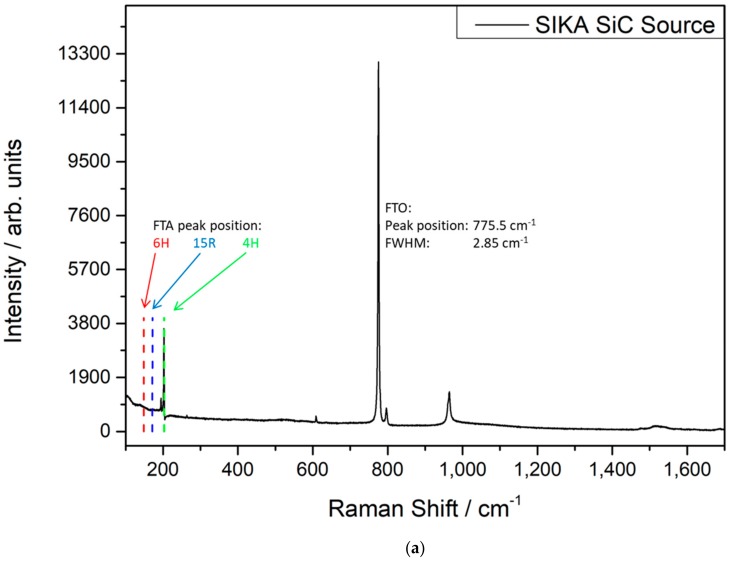

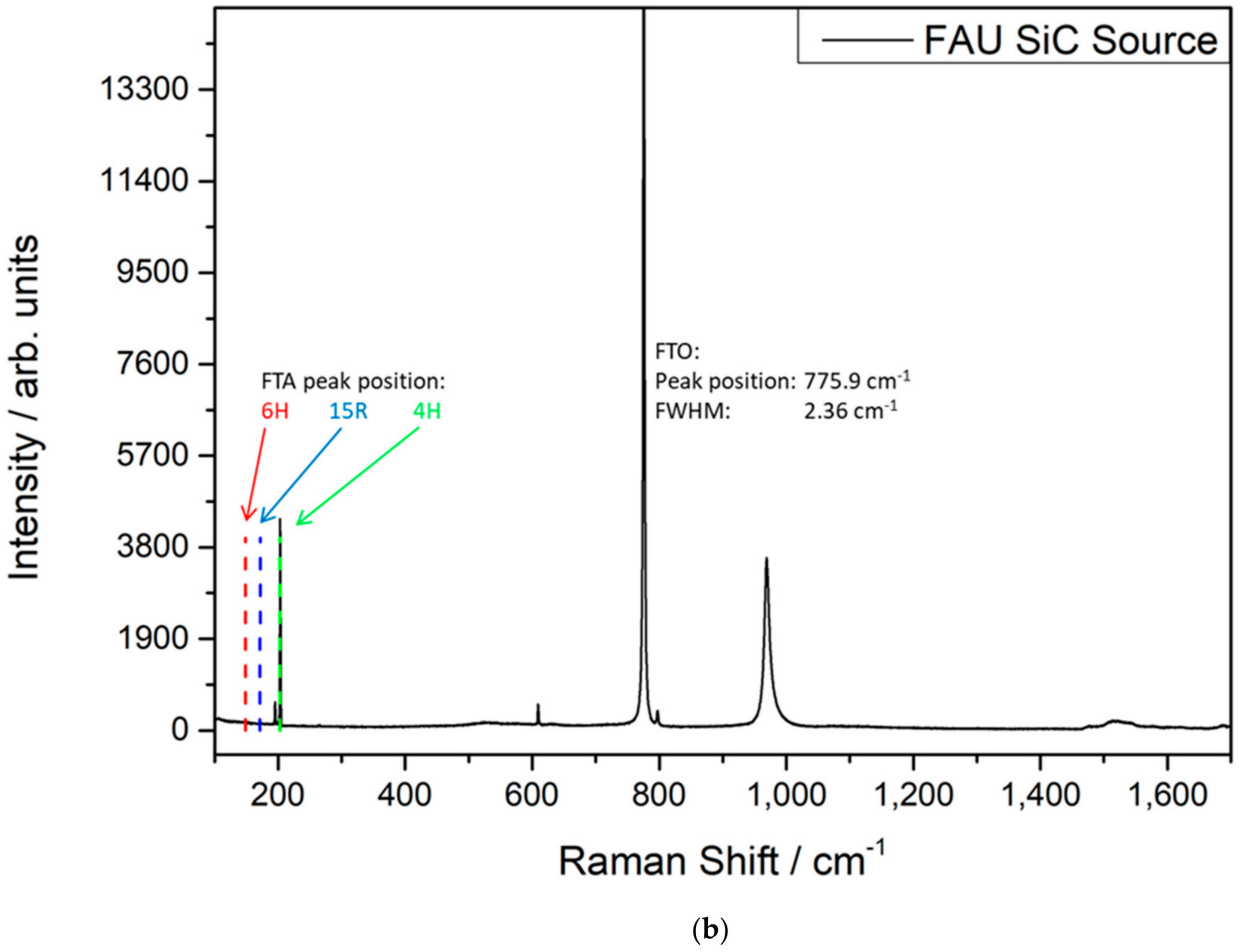
Raman spectra of wafers of the 75 mm SiC crystals grown with (**a**) the new SIKA and (**b**) the the FAU reference SiC powder.

In order to investigate the polytype stability during the application of the SIKA powder in the PVT system, Raman spectroscopy was carried out subsequent to the growth process on one wafer cut from the boule (Figure 4a). Solely a Raman signal related to the 4H-SiC polytype is observed, which is a proof for the stable PVT growth conditions using the SIKA powder. Compared to the reference crystal grown with the FAU SiC powder (Figure 4b), the position of the FTO line at ca. 776 cm^−1^ indicates comparable low stress values in the two crystals. The slightly smaller line width of the FTO line in the case of the FAU SiC source is related to a slightly higher SiC seed quality, but not to the applied SiC powder properties.

As an important conclusion, the morphology of the SIKA SiC powder does not release carbon dust particles at the sublimation interface that would interfere with the crystal growth process, causing the incorporation of macro-defects into the SiC boule. In addition, birefringence measurements did not indicate an increase of macro-defects in the SiC boule while using the SIKA instead of the FAU powder.

**Figure 5:**
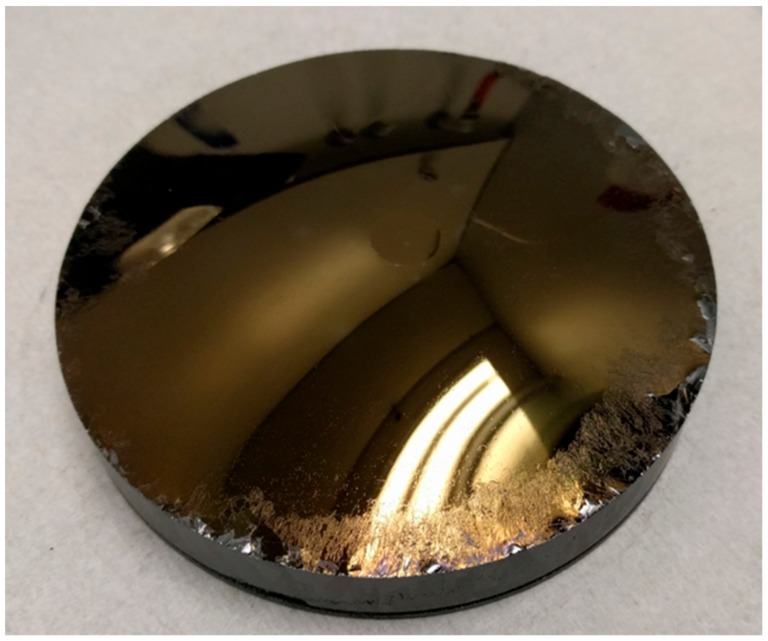
Optical image of the 100 mm SiC boule grown using the SIKA powder.

Another two SiC boules, however, with a single crystalline diameter of 100 mm were grown using the SIKA and FAU SiC powder source. The average growth temperature and ambient inert gas pressure were set to 2100 °C and 5 mbar, respectively. Nitrogen gas was added in order to perform n-type doping. Simultaneously to the growth process, in situ 2D X-ray imaging [9] was applied to study sublimation behavior in a setup that matches an industrial crystallization tool. Figure 5 shows an optical image of the top of the grown SiC boule which exhibits a predominantly mirror-like surface. 

**Figure 6:**
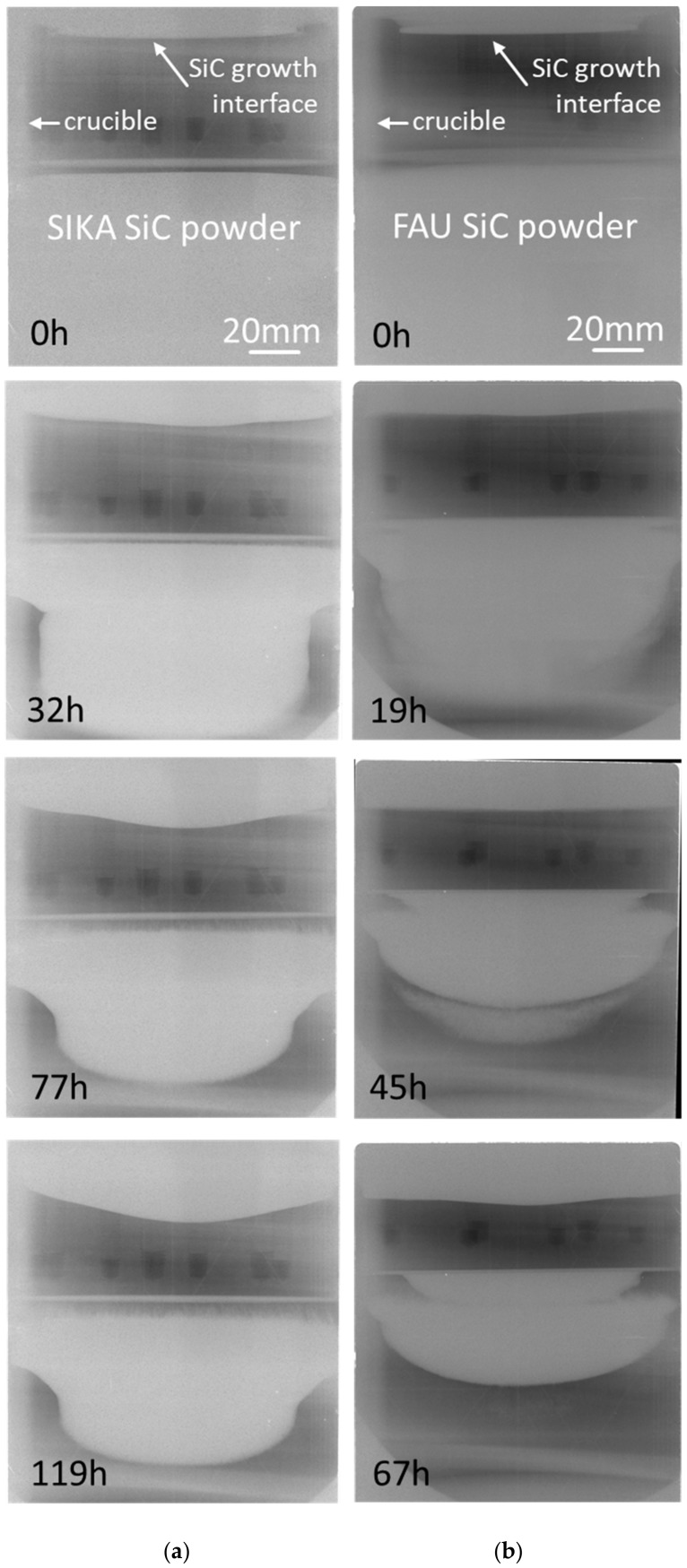
Series of 2D in situ X-ray visualization of the growth process of the 100 mm PVT growth process: (**a**) SiC powder from SIKA, and (**b**) reference SiC powder from FAU.

Figure 6 depicts for both growth runs, i.e., with SIKA and with FAU SiC powder, the evolution of the SiC source material and SiC crystal growth process. Similar to the case of the 75 mm SiC growth runs presented in Figure 3, the SIKA powder also exhibits a smoother sublimation-recrystallization behavior in the 100 mm SiC growth run than the FAU powder. As an example, in Figure 6, the SIKA SiC source exhibits one dense SiC powder block even after 77 and 119 h of growth. In the case of the FAU SiC source, however, the source separates in different SiC areas which are most pronounced after 45 and 67 h of growth (Figure 6). This observation is again to a large extent related to the difference in packing density of 1.73 g/cm^3^ (SIKA) vs. 1.44 g/cm^3^ (FAU). The crystal growth interface appears with a slightly more convex shape in the case of the SIKA powder compared to the FAU powder. As a high density in the powder area supports the formation of a temperature field with very low radial thermal gradients, the hot zone was adapted to introduce a stronger thermal gradient prior to the growth run with the SIKA powder. In the evolution of the growth run the curvature of the crystal is maintained steady over the growth period.

Based on the evolution of the growth process shown in Figure 5, the SIKA powder source material exhibits the properties for long term crystal growth runs. There is no visible gap between the crucible wall and the dense disk of the SIKA powder even after 119 h crystal growth while the FAU powder develops such a gap after only 45 h growth time. This enables much more constant growth conditions, e.g., the temperature field for long growth runs. In addition, very little morphology change in the images between 77 h and 119 h can be observed for the SIKA powder, indicating a very steady sublimation behavior for extended growth times. As a result, in the PVT setup used for the crystals as shown in Figure 6, the processing time using the SIKA source material could be prolonged to achieve a crystal height of 40 mm to 45 mm in the center.

## 4. Conclusions

The development of the new SiC powder source material, with an average particle size of ca. 300 µm and loosed packed density above 1.6 g/cm^3^, offers a number of properties beneficial for the growth of high quality SiC boules that is necessary for industrial application. The morphology of the SiC powder tends to suppress the release of carbon dust particles, which is advantageous in order to reach a high crystalline quality. The smooth sublimation behavior enables a homogeneous crystallization process exhibiting a stable, slightly convex SiC growth interface.

## Figures and Tables

**Table 1:** Summary of measured powder properties.

Property	Unit	Value
LPD	g/cm^3^	1.60
Free C	% wt	0.01
Total oxygen	% wt	0.02

**Table 2:** Typical chemistry of SIKA High Purity SiC powder as measured by GDMS as provided by the product sheet.

Element	Concentration (ppm wt)
Al	5.9
B	0.23
Ba	0.05
C	Matrix
Ca	0.67
Cl	1.5
Cr	0.41
Cu	0.18
F	<0.1
Fe	1.3
In	Binder
Na	0.62
Ni	0.33
P	0.13
Si	Matrix
S	1.6
Ti	2.8
V	0.19
Zn	0.45
Zr	0.22
